# Atherogenic L5 LDL induces cardiomyocyte apoptosis and inhibits K_ATP_ channels through CaMKII activation

**DOI:** 10.1186/s12944-020-01368-7

**Published:** 2020-08-21

**Authors:** Yanzhuo Ma, Nancy Cheng, Junping Sun, Jonathan Xuhai Lu, Shahrzad Abbasi, Geru Wu, An-Sheng Lee, Tatsuya Sawamura, Jie Cheng, Chu-Huang Chen, Yutao Xi

**Affiliations:** 1 Department of Cardiology, Bethune International Peace Hospital, 398 Zhongshan Xilu, Shijiazhuang, 050082 Hebei China; 2 Cardiac Electrophysiology Research Laboratory, Texas Heart Institute, 6770 Bertner Avenue, Houston, TX 77030 USA; 3 Vascular and Medicinal Research, Texas Heart Institute, 6770 Bertner Avenue, Houston, TX 77030 USA; 4 InVitro Cell Research, LLC, 106 Grand Avenue, Suite 290, Englewood, NJ 07631 USA; 5 Molecular Cardiology Research, Texas Heart Institute, 6770 Bertner Avenue, Houston, TX USA; 6 Department of Medicine, Mackay Medical College, No. 46, Section 3, Zhongzheng Road, Sanzhi District, New Taipei City, Taiwan 252; 7 Cardiovascular Research Laboratory, China Medical University Hospital, No. 2 Yude Road, North District, Taichung City, Taiwan; 8 Department of Life Innovation, Institute for Biomedical Sciences, Shinshu University, 3-1-1, Asahi, Matsumoto, Nagano, 390-8621 Japan; 9 Department of Molecular Pathophysiology, Shinshu University School of Medicine, 3 Chome-1-1 Asahi, Matsumoto, Nagano, 390-8621 Japan; 10 6770 Bertner Street, MC 2-255, Houston, TX 77030 USA

**Keywords:** Action potential, ATP-sensitive potassium, Ca^2+^/calmodulin-dependent protein kinase II, Cardiomyocytes, Electronegative low-density lipoprotein

## Abstract

**Background:**

Cardiac Ca^2+^/calmodulin-dependent protein kinase II (CaMKII) activation plays a critical role in cardiomyocyte (CM) apoptosis and arrhythmia. Functional ATP-sensitive potassium (K_ATP_) channels are essential for cardiac protection during ischemia. In cultured CMs, L5 low-density lipoprotein (LDL) induces apoptosis and QTc prolongation. L5 is a highly electronegative and atherogenic aberrant form of LDL, and its levels are significantly higher in patients with cardiovascular-related diseases. Here, the role of L5 in cardiac injury was studied by evaluating the effects of L5 on CaMKII activity and K_ATP_ channel physiology in CMs.

**Methods:**

Cultured neonatal rat CMs (NRCMs) were treated with a moderate concentration (ie, 7.5 μg/mL) of L5 or L1 (the least electronegative LDL subfraction). NRCMs were examined for apoptosis and viability, CaMKII activity, and the expression of phosphorylated CaMKIIδ and NOX2/gp91^phox^. The function of K_ATP_ and action potentials (APs) was analyzed by using the patch-clamp technique.

**Results:**

In NRCMs, L5 but not L1 significantly induced cell apoptosis and reduced cell viability. Furthermore, L5 decreased Kir6.2 expression by more than 50%. Patch-clamp analysis showed that L5 reduced the K_ATP_ current (I_KATP_) density induced by pinacidil, a K_ATP_ opener. The partial recovery of the inward potassium current during pinacidil washout was susceptible to subsequent inhibition by the I_KATP_ blocker glibenclamide. Suppression of I_KATP_ by L5 significantly prolonged the AP duration. L5 also significantly increased the activity of CaMKII, the phosphorylation of CaMKIIδ, and the expression of NOX2/gp91^phox^. L5-induced apoptosis was prevented by the addition of the CaMKII inhibitor KN93 and the reactive oxygen species scavenger Mn (III)TBAP.

**Conclusions:**

L5 but not L1 induces CM damage through the activation of the CaMKII pathway and increases arrhythmogenicity in CMs by modulating the AP duration. These results help to explain the harmful effects of L5 in cardiovascular-related disease.

## Background

Low-density lipoprotein (LDL) can be resolved into five charge-defined subfractions, L1 to L5. L1 is the least negatively charged and most abundant subfraction of circulating LDL and represents the healthy and harmless LDL class [1, 2]. L5 is the most negatively charged subfraction of circulating LDL and has been shown to induce cell apoptosis [3, 4]. Although L5 is nearly undetectable in healthy individuals, its concentrations are significantly higher in patients with cardiovascular-related diseases. Studies have indicated that L5 increases the incidence of thrombosis that contributes to ST-elevation myocardial infarction (STEMI) [5, 6] and that patients with STEMI have an increased risk of fatal arrhythmia [7]. In addition, L5 has been considered a novel factor for predicting coronary vascular disease [8] and stroke [9], and its concentrations are increased in individuals with cardiovascular risk factors. In cultured cardiomyocytes (CMs), L5 has been reported to induce cell apoptosis via cytokines released from vascular endothelial cells [10]. Recently, it has been demonstrated that L5 causes prolongation of the corrected QT interval (QTc) by mediating the current of L-type calcium and transient activated potassium channels [11]. Functional ATP-sensitive potassium channels (K_ATP_) are essential for ischemic preconditioning, post-ischemia cardiac protection, and regulation of atrial and ventricular rhythm during cardiac ischemia [12, 13]. However, the effect of L5 on K_ATP_ current (I_KATP_) has not been studied.

Cardiac Ca^2+^/calmodulin-dependent protein kinase II (CaMKII) is an underlying mechanism of CM apoptosis and arrhythmia [14, 15]. CaMKII oxidation serves as an intermediate of oxidative stress and correlates with increased apoptosis in cardiac cells [16]. In this study, the effects of L5 on the electrophysiologic properties of CMs were examined, as well as whether L5 induces CM damage through CaMKIIδ via the oxidation and phosphorylation of its regulatory domain.

## Methods

### L5 preparation and ion-exchange purification

Whole LDL was respectively collected from 10 patients with STEMI and metabolic syndrome, as described previously [1], with approval from the Institutional Review Board at the Texas Heart Institute (Houston, Texas, USA). Plasma samples were equilibrated by performing dialysis in a column loaded with buffer A (20 mmol/L Tris HCl [pH 8.0], 0.5 mmol/L EDTA, and 1% penicillin-streptomycin). Approximately 100 mg of LDL was injected onto a UnoQ12 anion-exchange column (Bio-Rad, Richmond, CA) by using a GE AKTA fast-protein liquid chromatography system (GE Healthcare Life Sciences, Logan, Utah, USA). LDL subfractions were eluted by using a multistep sodium chloride gradient, as previously described [17].

### Isolation of neonatal rat CMs (NRCMs)

NRCMs were isolated from neonatal Sprague Dawley rats [18]. Experiments were conducted according to The Code of Ethics of the World Medical Association (Declaration of Helsinki), and the animal procedures were approved by the Institutional Animal Care and Use Committee at the Texas Heart Institute (Houston, Texas, USA, protocol number #2011–18). Phenobarbital (10 mg/kg, administered intraperitoneally, Sigma-Aldrich Corp., St. Louis, MO, USA) was used to anesthetize the neonatal rats, and the left ventricle was quickly excised and rinsed three times with ice-cold Dulbecco’s phosphate-buffered saline (PBS; Invitrogen, Carlsbad, CA, USA). The ventricles were minced by using microdissection in a dry petri dish and then by enzymatic digestion with type II collagenase (Worthington Biochemical Co, Lakewood, NJ, USA) and procaine (Sigma-Aldrich Corp). After digestion, the cells were harvested and suspended in Dulbecco’s Modified Eagle’s medium (DMEM, Gibco, Carlsbad, CA, USA) with 10% fetal bovine serum (FBS; Invitrogen, Carlsbad, CA, USA), penicillin and streptomycin (100 U/mL, Invitrogen, Grand Island, NY, USA), and 2 mM glutamate (Invitrogen). Next, the cells were placed on 100-mm culture dishes, and the culture dishes were placed in a 37 °C incubator with 5% CO_2_. After fibroblasts settled, the supernatants were collected and replated for the following experiments.

### Cell apoptosis assays

NRCMs were starved overnight by using serum-free culture medium and were subjected to incubation with L5 or L1 for 24 h. Cell apoptosis was analyzed by using staining. Briefly, NRCMs (1.0 × 10^5^ cells/mL) were treated with different concentrations of L5 (0.075, 0.25, 0.75, 2.5, 7.5, 25, 75, or 250 μg/mL) overnight. Paraformaldehyde (5%) was used to fix the NRCMs. Then, the cells were incubated with Triton X-100 (0.1%) for 5 min. Next, the cells were stained with Hoechst (blue) for 15 min and were observed by using a fluorescence microscope. For Annexin V-Alexa 488/ propidium iodide (PI) staining (Invitrogen), NRCMs (1.0 × 10^5^ cells/mL) were placed in a 12-well culture plate and were treated with various concentrations of L5 (0.075, 0.25, 0.75, 2.5, 7.5, 25, 75, or 250 μg/mL) overnight. NRCMs were collected and rinsed with ice-cold PBS, followed by centrifugation and resuspension with 100 μL annexin binding buffer. Next, 5 μL Annexin V mixed with 1 μL PI (100 μg/mL) was added to the binding buffer. The cells were placed in the darkroom at room temperature. Then, 400 μL annexin binding buffer was added 15 min later, and cell apoptosis was measured by using a BD Biosciences imaging system. Cells staining positive for PI were excluded, and the ratio of Annexin V–positive cells to nuclei was quantified.

Apo-ONE Homogeneous Caspase-3/7 Assay (Promega, Madison, WI, USA) was used to examine cell apoptosis. Briefly, cells were plated on 96-well plates at a density of 10^4^ cells/well and were treated with media containing 100 μM H_2_O_2_, 7.5 μg/mL L5, 7.5 μg/mL L1, or PBS for 24 h. Cells were treated with reactive oxygen species (ROS) scavenger Mn (III) TBAP (Cayman Chemical, Ann Arbor, MI, USA) or CaMKII inhibitor KN93 (Sigma-Aldrich Corp) 30 min before L5 treatment or H_2_O_2_ treatment. Next, 100 μl of Apo-ONE® caspase-3/7 reagent containing caspase substrate and Apo-ONE® caspase-3/7 buffer was pipetted into each well. A plate shaker was used to mix the contents of the wells at 350 rpm for up to 1 h at room temperature. Fluorescence was measured at an excitation wavelength of 499 nm and an emission wavelength of 521 nm.

### Cell viability assay

NRCMs were plated on 96-well culture plates and were starved to allow synchronization. Next, NRCMs were incubated with 100 μM H_2_O_2_, 7.5 μg/mL L5, 7.5 μg/mL L1, or PBS for 24 h. As per the manufacturer’s instructions, MTT (Sigma-Aldrich Corp) was added at a concentration of 5 mg/mL, and cells were incubated in the dark. The liquid mixture was completely removed after 4 h. Then, cells were incubated with 150 μL dimethylsulfoxide for 10 min. The absorbance value was determined by using a spectrophotometer at 550 nm.

### Electrophysiologic recordings

Whole-cell current and voltage patch-clamp techniques were used for the I_KATP_ and action potential (AP) recording in NRCMs, as previously described [19]. Briefly, the currents were tested by using conventional techniques with an amplifier (Axopatch 700A) and software (pCLAMP Ver.9, Molecular Device, San Jose, CA, USA). Data were sampled and recorded by using an A/D converter (Digidata 1320, Molecular Device) with a Bessel 10-k*Hz* cut-off low-pass filter and a 10-k*Hz* sampling frequency. For the recording of APs, the intracellular pipette solution composition was as follows (in mM): 120 K-aspartate, 10 Na_2_ATP, 2 MgCl_2_, 10 EGTA, and 10 HEPES (pH 7.35 adjusted with KOH). The perforated patch was used, with the addition of amphotericin B (240 mg/L, Sigma-Aldrich Corp.) in the pipette solution. For the recording of the I_KATP_, the pipette solution composition was as follows (in mM): 120 K-aspartate, 0.5 Na_2_ATP, 2 MgCl_2_, 10 EGTA and 10 HEPES (pH 7.35 adjusted with KOH). Bath solution contained the following (in mmol/L): 135 NaCl, 1 MgCl_2_, 1.8 CaCl_2_, 5.4 KCl, 10 HEPES, and 10 glucose (pH 7.35 adjusted with NaOH). Calcium current blocker, nifedipine (2 nM, Sigma), Glibenclamide transient potassium current blocker, and 4-aminopyridine (2 mmol/L, Sigma-Aldrich Corp) were added. A − 40 mV prepulse was used to deactivate sodium currents. Voltage steps of 100 ms from potentials of − 100 to 60 mV were applied at 0.5 *Hz* in 10-mV increments. NRCMs with clear structure were studied.

### Cell lysis and Western blotting analysis

Culture medium was removed after different treatments. NRCMs were washed with ice-cold PBS, and the PBS was completely removed by tapping the plate onto a sheet of absorbent paper. Radioimmunoprecipitation assay (RIPA) lysis buffer with protease and phosphatase inhibitors was added to the cells, and the plate was placed on ice until the cells had a round appearance. Culture medium was added to the plate to terminate cell lysis. Next, a pipette was used to dissociate the cells until most cells had fallen off, and the lysate was then transferred to an Eppendorf tube. Cells were centrifuged for 15 min at 12,000 rpm to obtain protein. All steps were performed at 4 °C. The protein was denatured by boiling the cell lysate for 3 min at 100 °C.

Proteins were loaded and electrophoresed on sodium dodecyl sulfate polyacrylamide gels and were then transferred to polyvinyledine fluoride (PVDF) filter membranes. Monoclonal antibodies against Kir6.2 (rabbit polyclonal, 1:200; Alomone Labs Ltd, Jerusalem, Israel), Nox2/gp91phox (rabbit polyclonal, 1:1000, Abcam, Cambridge, United Kingdom), or CaMKIIδ (rabbit polyclonal, 1:200; Santa Cruz Biotechnology, Santa Cruz, California, USA) were used for immunoblotting overnight at 4 °C. PVDF membranes were incubated for 1 h with horse radish peroxidase (HRP)-conjugated anti-rabbit or mouse immunoglobulin G antibody (1:5000, Santa Cruz Biotechnology) at room temperature. Kodak films were used to record signals. The band densities were analyzed by using Image J software.

### CaMKII activity assay

CaMKII activity was examined by using a CaMKII enzyme-linked immunosorbent assay (CycLex Co, Nagano, Japan). The samples and positive control (10 μL each) were pipetted onto a 96-well microtiter plate. Then, 90 μL kinase reaction buffer was incubated with the cells and the positive control at 30 °C, followed by an incubation at room temperature for 30 min. Each well was rinsed five times with wash buffer. Next, the cells were incubated with 100 μL HRP-conjugated antibody with the plate sealed for 60 min. Each well was rinsed with wash buffer again, and the cells were incubated with 100 μL substrate reagent for 10 min. Then, 100 μL stop solution was added to each well. By using a spectrophotometric plate reader, absorbance was measured at dual wavelengths of 450 and 550.

### Statistical analysis

Western blotting and ELISA data were expressed as the mean ± standard deviation, and patch-clamp data were expressed as the mean ± standard error of the mean. All data were subjected to analysis of variance and then Dunnett’s test. The normality of data distribution was tested by using the Shapiro–Wilk test. A *P*-value less than 0.05 was considered statistically significant.

## Results

### L5 induces cell injury in NRCMs

**Fig. 1:**
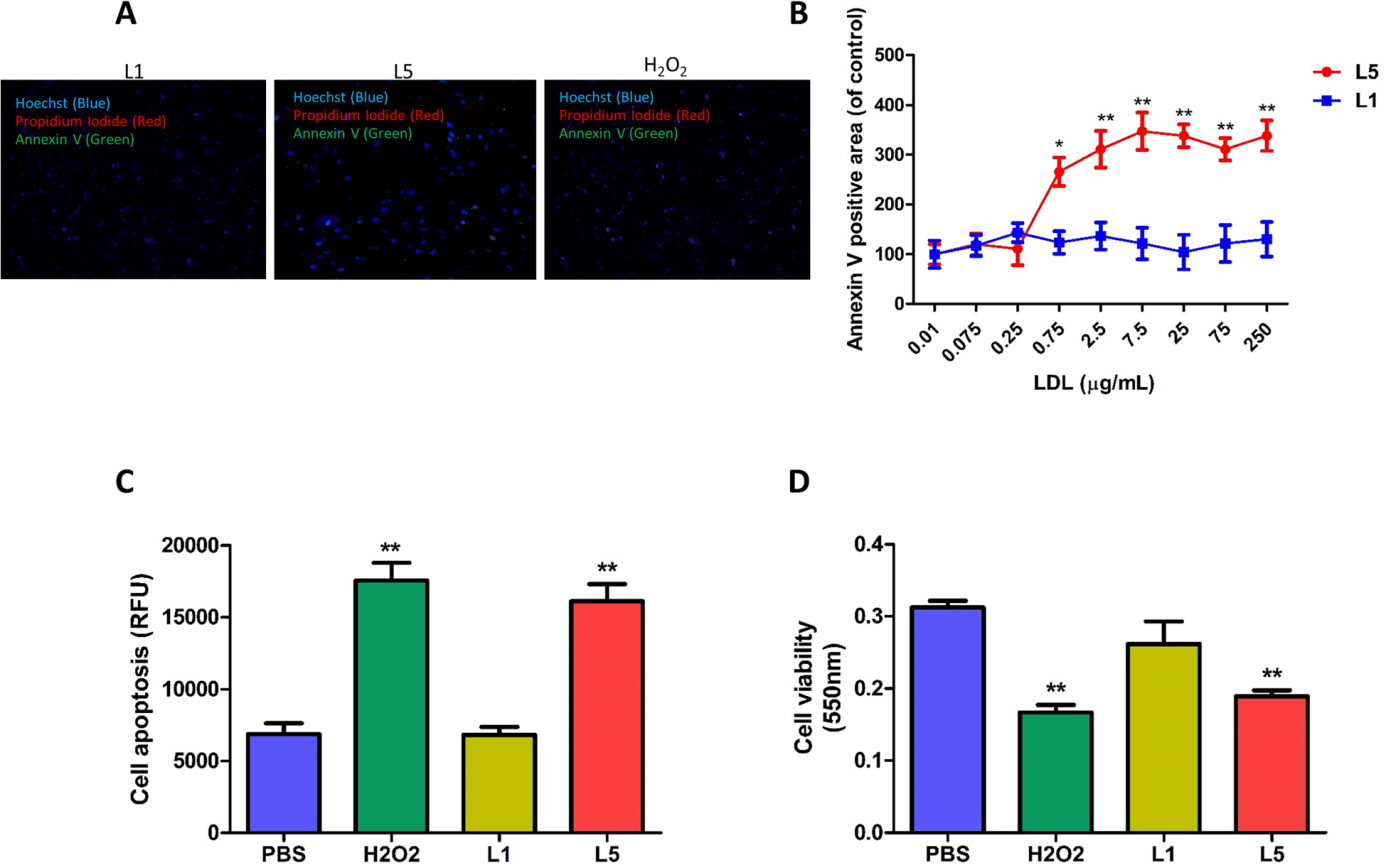
Increased apoptosis and decreased cell viability in NRCMs treated with L5. **a**, Early apoptosis of NRCMs treated with L5, shown by representative images of Hoechst (blue, nuclei), Annexin V-Alexa 488 (green), and PI (red) staining. **b**, Dose-dependent curve showing the effects of increasing concentrations of L5 and L1 on the percentage of cells staining positive for Annexin V (*n* = 6 per group). **c**, Relative apoptosis levels of NRCMs after the indicated treatment, determined by using a caspase-3/7 activity assay (*n* = 7 per group). **d**, Cardiomyocyte viability was analyzed after the indicated treatment by using an MTT kit, n = 6 per group. *P < 0.05, **P < 0.01 vs. the PBS-treated group

Cell apoptosis was examined in NRCMs treated with L5 or L1 (control) by using Hoechst staining (blue, nuclei), Annexin V-Alexa 488 (green), and PI (red) staining. In L5-treated cells, only a few were stained by PI, which indicated the early apoptosis of NRCMs (Fig. 1a). In contrast, L1 showed a negligible effect on apoptosis. The number of Annexin V–positive cells was markedly increased as the L5 concentration increased from 0.75 up to 7.5 μg/mL, the concentration at which L5’s effects reached a plateau. No significant change in Annexin V–positive cell number was observed when NRCMs were incubated with L1 at any concentration (Fig. 1b). Furthermore, compared with L1 or PBS, L5 markedly enhanced the activity of caspase-3/7 protease (Fig. 1c) and significantly decreased cell viability (Fig. 1d).

### L5 prolongs action potential duration (APD) in NRCMs

**Fig. 2:**
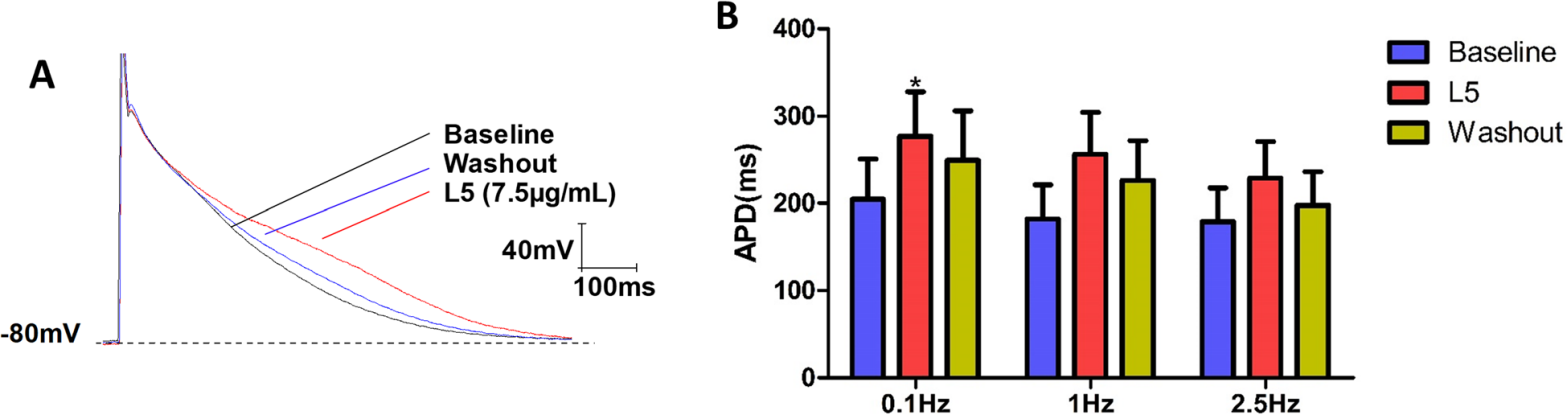
Prolonged APD75 in NRCMs after L5 treatment. **a**, Representative traces of the APD75 in NRCMs treated with L5. **b**, Comparison of the APD75 at 0.1 *Hz*, 1 *Hz*, and 2.5 *Hz*. *n* = 5 per group. *P < 0.05 vs. baseline

In CMs, L5 has recently been shown to induce abnormal electrophysiologic activities, such as prolonged action potential duration (APD) [11]. In this study, L5 significantly prolonged the APD in NRCMs (Fig. 2a). In addition, the APD at 75% repolarization (APD75) was more prolonged in NRCMs treated with L5 than in untreated NRCMs at 0.1 *Hz* frequency (Fig. 2b). The L5-induced prolongation of APD75 occurred 5 to 10 min after treatment but did not fully recover until after the 30-min washout.

### L5 downregulates K_ATP_ channel expression and function

**Fig. 3:**
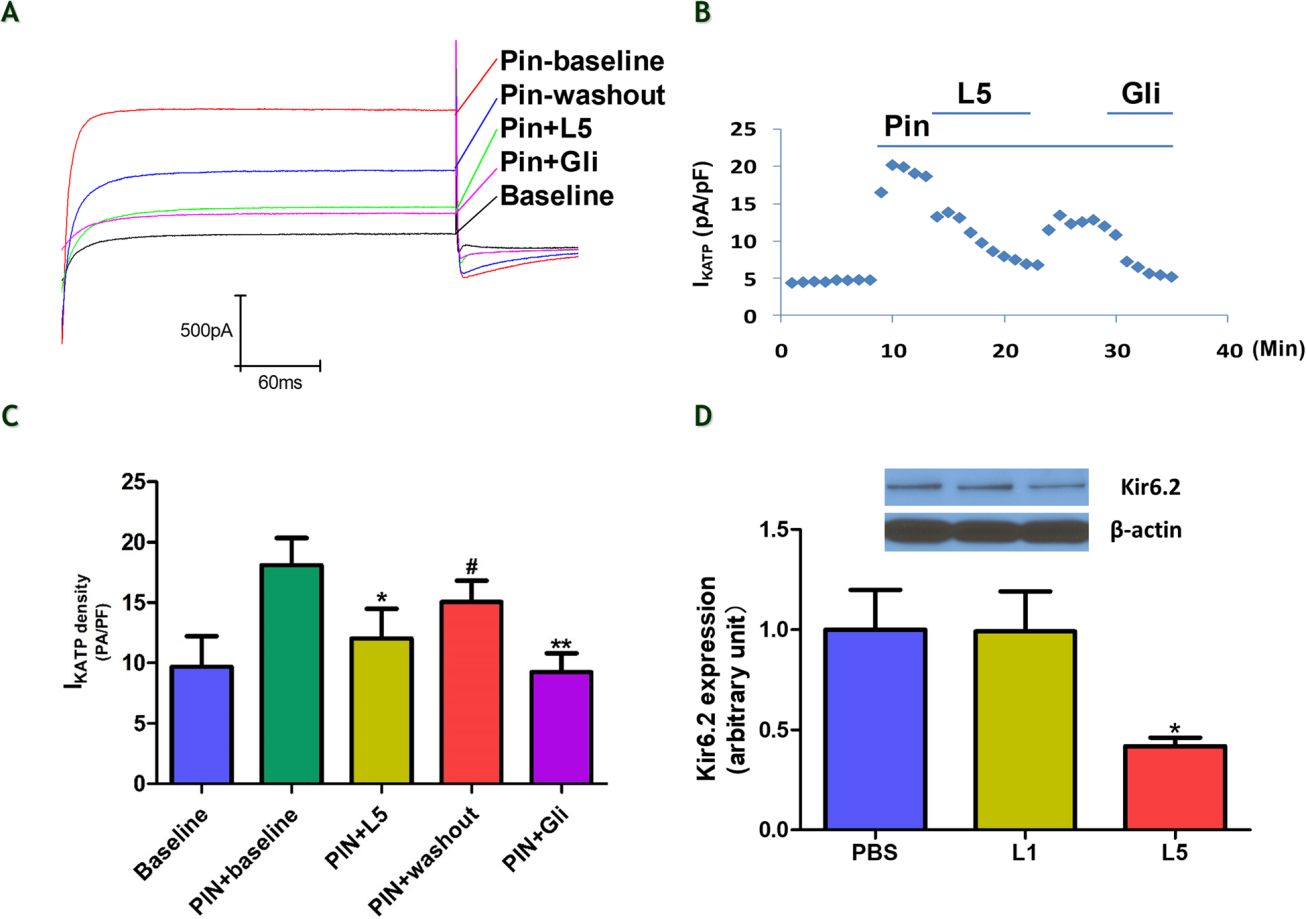
Decreased I_KATP_ density in NRCMs treated with L5. **a**, Representative traces of I_KATP_ in NRCMs treated with Pin-baseline (20 μM pinacidil, IKATP opener), Pin+L5 (7.5 μg/mL), Pin-washout, and Pin+Gli (100 μM glibenclamide, I_KATP_ blocker). **b**, Time-course analysis showing the effect of L5 on I_KATP_. **c**, The perfusion of NRCMs with L5 reduced I_KATP_, which partially recovered after 5 min of washout (n = 5 per group, **P < 0.01,*P < 0.05 vs. Pin-baseline, #*P* < 0.05 vs. Pin+L5). **d**, The overnight incubation of NRCMs with L5 decreased the expression level of Kir6.2, but no change was seen after overnight incubation with L1, *n* = 3 per group. *P < 0.05 vs. PBS

The effect of L5 on I_KATP_ was examined in the presence of the I_KATP_ opener pinacidil (Pin; 20 μM). Figure 3a shows representative traces of I_KATP_ in the following groups of treated NRCMs: Pin-baseline, Pin+L5 (7.5 μg/mL), Pin-washout, and Pin+glibenclamide (Gli; 100 μM, I_KATP_ blocker). The perfusion of L5 decreased the level of Pin-opened I_KATP_, reaching its maximum effect in about 8 min. This effect of L5 on I_KATP_ was then partially recovered after 5 min of washout. After the washout, the addition of the Kir6.2-specific blocker Gli attenuated the level of Pin-opened I_KATP_ 5 min after treatment, similar to L5 (Fig. 3b, c). In addition, L5 but not L1 decreased the expression of Kir6.2 by more than 50% when compared with PBS (Fig. 3d).

### Reactive oxygen species (ROS) and L5 increase CaMKII activity and NOX2/gp91^phox^ expression in NRCMs

**Fig. 4:**
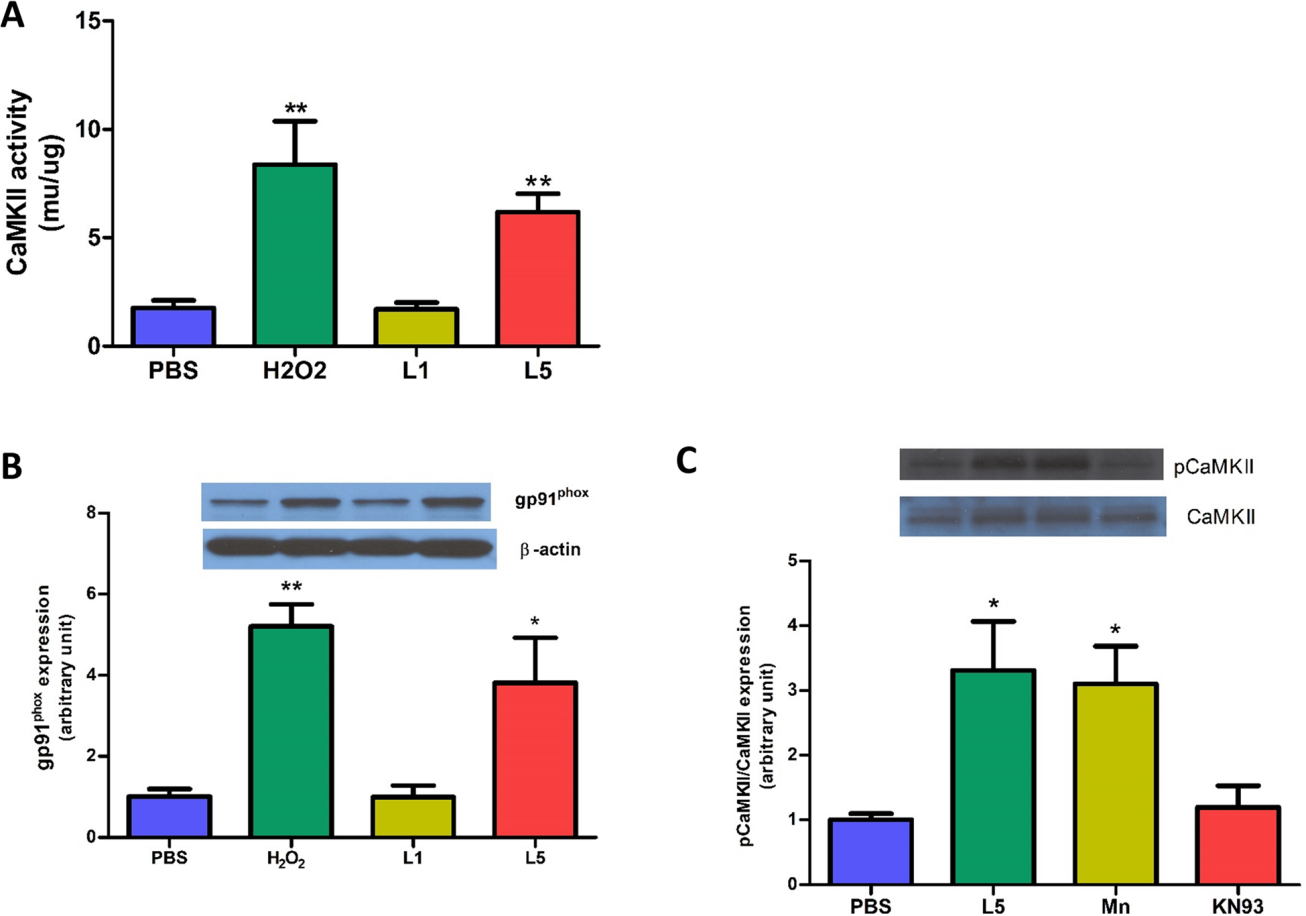
Increased CaMKII activity and ROS production in NRCMs treated with L5. **a**, L5 increased CaMKII activity in NRCMs, whereas L1 did not (*n* = 8 per group). **b**, L5 increased the expression level of NOX2/gp91phox in NRCMs, whereas L1 did not (n = 3 per group). **c**, L5 induced CaMKII phosphorylation, and the CaMKII inhibitor KN93 decreased L5-induced CaMKII phosphorylation. The ROS scavenger Mn (III) TBAP did not affect L5-induced CaMKII phosphorylation (n = 3 per group). *P < 0.05, **P < 0.01 vs. PBS

Compared with PBS (control), L5 significantly increased CaMKII activity in NRCMs, whereas L1 did not (Fig. 4a). Similar to L5, H_2_O_2_ also markedly increased CaMKII activity (Fig. 4a). Next, the effect of L5 on the expression of NOX2/gp91^phox^ was examined. Compared with PBS, L5 increased the expression of NOX2/gp91^phox^ in NRCMs, an effect that was similar to that of H_2_O_2_ (positive control; Fig. 4b), whereas L1 did not. L5 also increased the phosphorylation of CaMKIIδ in NRCMs (Fig. 4c). The addition of the CaMKII blocker KN93 attenuated the L5-induced increase in the phosphorylation CaMKII, but the ROS scavenger Mn (III) TBAP did not (Fig. 4c).

### The inhibition of ROS and CaMKII pathways prevents cell injury induced by L5

**Fig. 5:**
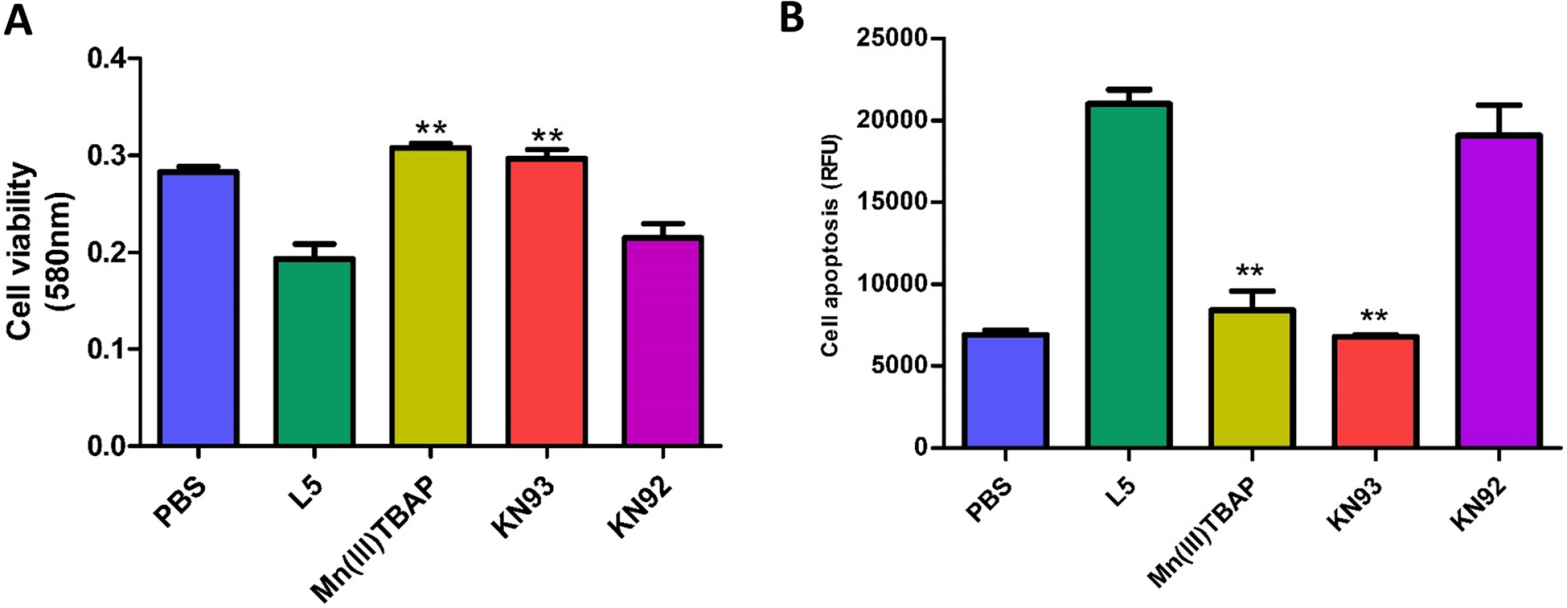
The attenuation of L5’s effect on NRCMs by inhibitors of ROS and CaMKII. **a**, The ROS scavenger Mn (III) TBAP and CaMKII inhibitor KN93 prevented the L5-induced decrease in the cell viability of NRCMs. **b**, Mn (III) TBAP and KN93 attenuated the increased apoptosis of NRCMs caused by L5. n = 6 per group. **P < 0.01 vs. L5

Exposure to L5 significantly decreased cell viability, whereas Mn (III) TBAP (ROS scavenger) and KN93 (CaMKII inhibitor) prevented the significant L5-induced decrease in cell viability (Fig. 5a). Similarly, L5 increased the percentage of apoptotic cells, whereas Mn (III) TBAP and KN93 reduced the apoptosis of NRCMs treated with L5 (Fig. 5b). These results indicated that L5 induces NRCM injury via CaMKII and ROS pathways.

## Discussion

Plasma LDL plays a critical role in the pathogenesis of coronary heart disease (CHD) and in the treatment of CHD [20]. Recently, it has been demonstrated that electronegative LDL is atherogenic and exerts deleterious effects that lead to the development of CHD [21]. Furthermore, electronegative LDL is considered to be a novel risk factor for CHD. The most electronegative subfraction of LDL—L5 —has been shown to have atherogenic functions in vitro and in vivo. Clinical trials have been performed to evaluate the correlation between increased L5 concentrations and atherosclerosis risk in patients with CHD or other diseases. These studies have shown the predictive power of L5 levels and that an increased L5 level is positively correlated with atherosclerosis in patients with CHD or hyperlipidemia [22, 23]. In addition, an increased L5 level may lead to early-stage vessel aging in patients with systemic lupus erythematosus [24] and increases the chance of developing atherosclerosis in patients with rheumatoid arthritis [25]. The underlying mechanisms of L5-induced atherogenicity include mitochondrial dysfunction and premature endothelial senescence [26]; increased production of granulocyte colony-stimulating factor in macrophages [27]; the promotion of macrophage maturation and infiltration; and the resultant adipose inflammation [28]. In this study, L5 but not L1 induced early cell apoptosis in NRCMs. Furthermore, L5-induced cell damage was attenuated by the ROS scavenger Mn (III) TBAP and the CaMKII inhibitor KN93, revealing that L5 may act through the ROS and CaMKII pathways. These results strongly suggest that L5 is detrimental to NRCMs and provide new insights into the underlying mechanism of L5’s harmful effects on NRCMs.

Ion channels are a critical determinant for AP shaping, which modulates regional electrophysiologic properties and has a complex role in cardiac arrhythmias [29]. A variety of proteins can regulate ion channels and the AP to develop a pro-arrhythmic effect [30, 31]. Chang et al. [32] previously showed that cholesterol levels are associated with QTc dispersion. In addition, L5 levels have been shown to correlate with the prolonged duration of QTc intervals in patients with CHD. This prolongation was modulated by the alteration of the I_CaL_ (L-type calcium current) and I_To_ (transient outward potassium current) [11]. Dysfunctional potassium channels primarily affect cardiac conduction, as evidenced by altered PR and QTc intervals, as well as APD alternans. A variety of potassium channels, such as inward rectifier potassium channel current [33], delayed rectifier potassium channels (I_Kr_ and I_Ks_) [34, 35], and outward-rectifying potassium current [36], have been well studied and are responsible for APD variation. K_ATP_ channels are also important contributors to overall cardiac electrophysiology and arrhythmias [37]. In this study, L5 induced the prolongation of APD and increased I_KATP_ density, implying that L5 may induce arrhythmias. K_ATP_ channels can be activated under ischemic conditions or directly by pinacidil [38]. K_ATP_ consists of Kir6.1 or Kir6.2 and an ATP-binding subunit (eg, SUR1, SUR2A, or SUR2B) [39]. SUR2A and Kir6.2 are major constituents of mouse and rat hearts, and Kir6.2 is required for cardioprotection [40]. Here, L5 but not L1 decreased the expression of Kir6.2 when incubated with NRCMs overnight, indicating that the downregulation of K_ATP_ current is not only caused by channel abundance but also by channel modification.

CaMKII is a protein kinase with key roles in various cardiac diseases, including heart failure, acute myocardial infarction, and malignant arrhythmias [41, 42]. CaMKII consists of four isoforms: α, β, γ, and δ. Of those, the γ and δ isoforms are predominantly expressed in the heart. In addition, CaMKII activity is primarily regulated by four posttranslational modifications, including the Ca^2+^/calmodulin-binding, autophosphorylation [43, 44], oxidation [45], O-linked N-acetylglucosamination (O-GlcNAc) [46], and S-nitrosylation [47] of CaMKII. Several studies have confirmed that the sustained activation of CaMKII modulates potassium channels [48], whereas the inhibition of CaMKII may prevent the internalization of K_ATP_ channels caused by ischemia, thereby reducing the vulnerability of CMs to injury [49, 50]. This effect was further confirmed by the attenuation of L5-induced cell injury by a CaMKII inhibitor. Recently, CaMKII has been identified as an ROS sensor in the heart [51]. In this study, H_2_O_2_ mimicked the effect of L5 on CaMKII activity, suggesting that ROS act upstream of CaMKII. However, the effect of L5 on CaMKII phosphorylation was not affected by a ROS scavenger, indicating that ROS act only on the oxidative posttranslational modifications of CaMKII and that autophosphorylation and oxidation are the two main mechanisms of CaMKII activation. These results suggested that L5 exerted its effect through the phosphorylative and oxidative modification of CaMKIIδ.

### Study strengths and limitations

In this study of the direct effects of electronegative LDL on the properties of cardiomyocytes, evidence is presented showing how electronegative LDL injures CMs, suggesting potential mechanisms for how high levels of L5 in patients with STEMI relate to an increased risk of fatal arrhythmia. A limitation of this study is that, because of the low amount of L5 obtained from each study participant, the L5 samples are pooled from multiple patients. This may result in the data reflecting an averaged effect of L5 on CMs.

## Conclusions

In conclusion, these study findings indicate that L5 is detrimental to CMs and may critically increase cell damage in patients at risk for or who have cardiovascular events. Moreover, L5 may serve as a risk factor for the detection of CHD and its related diseases. Additional studies are warranted to further identify the clinical significance of L5 in cardiovascular-related injury.

## Data Availability

Additional data and materials can be obtained by contacting the corresponding author.

## Abbreviations

APD: Action potential duration
CaMKII: Ca2+/calmodulin-dependent protein kinase II
CHD: Coronary heart disease
CM: Cardiomyocyte
LDL: Low-density lipoprotein
I_KATP_: ATP-sensitive potassium channel current
K_ATP_: ATP-sensitive potassium channel
PBS: Phosphate-buffered saline
ROS: Reactive oxygen species
STEMI: ST-elevation myocardial infarction
